# Microbiota Dysbiosis in Fungal Rhinosinusitis

**DOI:** 10.3390/jcm8111973

**Published:** 2019-11-14

**Authors:** Yen-Ting Lu, Shao-Hung Wang, Ming-Li Liou, Ting-An Shen, Ying-Chou Lu, Chung-Han Hsin, Shun-Fa Yang, Yih-Yuan Chen, Tzu-Hao Chang

**Affiliations:** 1 Department of Otolaryngology, St. Martin De Porres Hospital, Chiayi 600, Taiwan; ytlu.tw@gmail.com (Y.-T.L.); luyingchou@gmail.com (Y.-C.L.); 2 Department of Otolaryngology, Chung Shan Medical University Hospital, Taichung 402, Taiwan; Hsin@csmu.edu.tw; 3 Institute of Medicine, Chung Shan Medical University, Taichung 402, Taiwan; ysf@csmu.edu.tw; 4 Department of Microbiology, Immunology and Biopharmaceuticals, National Chiayi University, Chiayi 600, Taiwan; shwang@mail.ncyu.edu.tw; 5 Department of Medical Laboratory Science and Biotechnology, Yuanpei University, Hsin-Chu City 300, Taiwan; d918229@gmail.com; 6 Graduate Institute of Biomedical Informatics, Taipei Medical University, Taipei City 110, Taiwan; aria607048@gmail.com; 7 Department of Medical Research, Chung Shan Medical University Hospital, Taichung 402, Taiwan; 8 Department of Biochemical Science and Technology, National Chiayi University, Chiayi 600, Taiwan; 9 Clinical Big Data Research Center, Taipei Medical University Hospital, Taipei City 110, Taiwan

**Keywords:** chronic rhinosinusitis, microbiota, dysbiosis, fungal rhinosinusitis, non-fungal rhinosinusitis, microbiome, next-generation sequencing, 16srRNA

## Abstract

Fungal rhinosinusitis is a unique phenotype of chronic rhinosinusitis with unique clinical and histological characteristics. The role of bacterial microbiota in various phenotypes chronic rhinosinusitis is not thoroughly understood. Therefore, we conducted 16s rRNA amplification sequencing to determine differences in bacterial communities between phenotypes (fungal vs. non- fungal) and anatomical sites (middle meatus vs. nasopharynx). Endoscope-guided swabs were used to collect samples from the middle meatus and nasopharynx of seven consecutive patients with fungal and 18 consecutive patients with non-fungal rhinosinusitis. DNA was extracted and investigated through 16S rRNA amplification. Among samples from the middle meatus, Shannon diversity was significantly lower in those from the fungal rhinosinusitis group (*p* = 0.029). However, no significant differences in diversity were noted between nasopharynx samples (*p* = 0.85). Fungal rhinosinusitis samples exhibited a distinct distribution of taxon relative abundance, which involved not only the absence of rhinosinusitis-associated commensal *Corynebacterium* and *Fusobacterium* in the middle meatus but also a significant increase in *Haemophilus* prevalence and abundance. This is the first study to compare bacterial communities in fungal and non-fungal rhinosinusitis samples. Our findings demonstrated that bacterial community dysbiosis was more apparent in fungal rhinosinusitis samples and was limited to the middle meatus.

## 1. Introduction

Rhinosinusitis is a common inflammatory disease of the sino-nasal cavity. Host and environmental factors both play a key role in rhinosinusitis development [1]. Among possible environmental factors, the local microbiome is a major factor influencing rhinosinusitis development. Increasing numbers of studies, particularly after the development of culture-independent sequencing techniques, have discussed the association between the nasal microbiome and rhinosinusitis. Conventional culture techniques provide only limited evidence regarding bacterial communities; by contrast, next-generation sequencing (NGS), a culture-independent technique, is a superior representation of resident microbiota [2]. A study on samples obtained from 54 patients with chronic rhinosinusitis (CRS) reported that compared with 16S sequencing, standard clinical culture data revealed as low as 47.7% of taxa in the samples [2]. Culture-independent sequencing could detect bacterial community dysbiosis in samples obtained from patients with CRS; dysbiosis may be an additive factor to the severity of CRS [3]. Hoggard et al. included 94 patients with bilateral CRS and 29 health controls in their study, and they observed aberrant (dysbiotic) bacterial assemblages dominated by *Corynebacterium* in the samples from the patients with CRS [3].

Fungal rhinosinusitis, a particular phenotype of rhinosinusitis, has a specific clinical presentation that is different from non-fungal sinusitis. However, despite advances in techniques for the characterization of bacterial communities in patients with CRS, research on the fungal micro- ecology or mycobiome in patients with CRS is lacking [4]. Zhao et al. conducted internal transcribed spacer (ITS) sequencing and reported that fungal dysbiosis occurred in only selected patients with CRS; this could thus not serve as a universal determinant of sinus disease pathogenesis [4]. Further research applying NGS to evaluate bacterial microbiomes or communities and their association with fungal and non-fungal rhinosinusitis is necessary.

The aim of the present study was to analyze and compare the microbiomes in patients with fungal rhinosinusitis and those with non-fungal rhinosinusitis (control group) through 16S rRNA sequencing in order to elucidate the microbial difference between the two phenotypes of rhinosinusitis and the possible interaction between fungi and bacteria.

## 2. Material and Methods

### 2.1. Study Design and Population

In this prospective cohort study, all patients were consecutively recruited from St. Martin De Porres Hospital, Taiwan. Approval for the study was obtained from the Human Ethics Committee of this institute (No.17B-006).

Patients (aged ≥ 20 years) were enrolled if they were diagnosed as having CRS as defined by the American Rhinology Society and underwent functional endoscopic sinus surgery [5]. Patients who received topical or systemic antibiotics/antifungal medication within one month before surgery, had sinonasal malignancy, reported pregnancy, had cystic fibrosis, or were immunocompromised were excluded from the study [4]. Patients were subgrouped into a fungal or non-fungal rhinosinusitis group based on the microscopic presence or absence of fungal hyphae in the pathological findings, respectively. Demographic data and endoscopic and radiologic findings were also recorded.

### 2.2. Sample Collection

Samples were collected using endoscopy-guided swabs during functional endoscopic sinus surgery at St. Martin De Porres Hospital. All surgical and sample collection procedures were performed by the same surgeon (Y-T.L.). Cultures for DNA extraction were obtained from two locations—namely (1) the middle meatus of the sinusitis lesion site and (2) the nasopharynx—by applying a swab to the surface and rotating it for at least five full turns until it became visibly saturated [6]. The inferior turbinate was laterized with elevator temporarily, and the nasopharynx was sampled carefully to avoid swab contamination [6]. All swabs were placed in a sterile container on ice for immediate transport to the laboratory and were subsequently stored at −80 °C until DNA extraction. Bacterial culture and pathological specimens were also collected from individual patients and analyzed. In addition to routine histological staining, silver staining was arranged for the pathological specimens. In this study, “fungal rhinosinusitis” was defined as (1) presence of fungal hyphae and (2) positive silver staining results for the pathological specimen, as observed under a microscope by a pathologist.

### 2.3. DNA Extraction

Microbes were collected using sterile swabs. After the sampling procedure, DNA extraction was performed using the Qiagene Blood Mini Kit (Qiagene) according to the manufacturer’s instructions. The resultant DNA quality and quantity were measured using 1% agarose gel electrophoresis and a Nanadrop ND1000 spectrophotometer (Thermo Fisher Scientific, Waltham, MA).

### 2.4. 16S Metagenomics Sequencing 

Polymerase chain reaction (PCR) amplification was performed using the V3 forward primer 5′CCTACGGGGNGGCWGCAG-3′ and V4 reverse primer 5′GACTACHVGGGTATCTAATCC-3′, producing a 300-bp amplicon spanning the highly variable V3–V4 region of the 16S rRNA gene. Paired-end sequence data in FASTQ format were obtained using the Illumina platform, and a FASTX- Toolkit was used to assess sequence quality. Raw reads were demultiplexed by barcodes, and adaptor sequences were removed. A minimum Phred quality score (Q score) of 30 was applied to trim low-quality bases.

### 2.5. Microbial Community Analysis and Statistical Analysis

QIIME2 was applied for alpha diversity, beta diversity, and principal coordinate analyses (PCoA) using the Bray–Curtis distance. DESeq2 in QIIME was used to identify operational taxonomic units (OTUs) that differed between phenotypes (fungal vs. non-fungal) and anatomical sites (middle meatus vs. nasopharynx). The prevalence of a specific taxon was calculated by the number of samples containing the taxon divided by the total number of samples. Co-occurring network correlations were computed on the basis of a pairwise Pearson correlation analysis in the R language. Cytoscape, which is a bioinformatics software platform for visualizing molecular interaction networks and integrating with gene expression profiles, was also used to draw a genus network. Correlations with a Pearson correlation coefficient greater than 0.6 were transformed to links between two genera in the genus network. Taxa with a significant difference between the fungal and nonfungal rhinosinusitis samples were collected to analyze the overlap between different anatomical sites of nasal samples.

## 3. Results

This study included 25 consecutive patients with rhinosinusitis. Table 1 presents the demographic data of these patients. Among these patients, seven were assigned to the fungal rhinosinusitis subgroup because the presentation of fungal hyphae was confirmed by their histopathology reports. PCR revealed that the fungal hyphae belonged to *Aspergillus.* However, fungal hyphae were discovered only in the middle meatus samples obtained from patients in the fungal rhinosinusitis subgroup. Moreover, nasopharynx samples obtained from the patients in the fungal rhinosinusitis group presented no fungal hyphae. Among clinical features, the extension of fungal rhinosinusitis was restrictive comparing with non-fungal rhinosinusitis. All fungal rhinosinusitis were unilateral and limited in isolated maxillary sinus, but nine of 18 of non-fungal rhinosinusitis cases presented as bilateral lesion and all of them with multi-sinus inflammation.

A total of 48 nasal samples (from the middle meatus and nasopharynx) were collected from the patients: 13 fungal rhinosinusitis samples (from seven patients with fungal rhinosinusitis) and 35 nonfungal rhinosinusitis samples (from 18 patients with non-fungal rhinosinusitis). After demultiplexing and quality control assessments, a total of 1,318,910 sequence reads were obtained from the samples, with a median of 27,725 reads and a median read length of 343 bp per sample. After microbial taxonomy assignment, a mean of 25,239 OTU-mapped reads and 128 OTUs were obtained per sample. The OTUs at the genus level in both the middle nasal meatus and nasopharyngeal samples are provided in Supplementary File 1.

**Figure 1:**
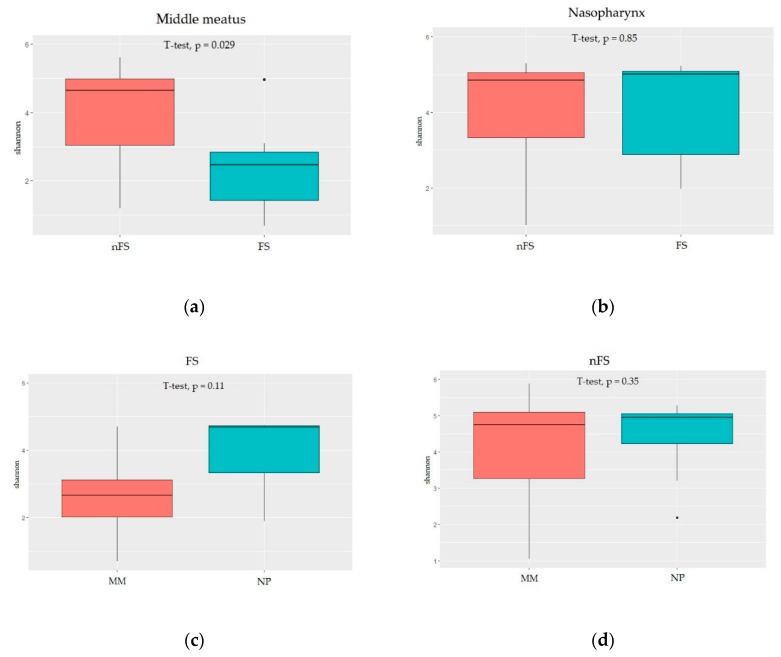
Differences in Shannon diversity (alpha diversity) between samples collected from different anatomical sites in fungal and non-fungal rhinosinusitis groups. (**a**) Middle meatus (lesion site) samples from the fungal rhinosinusitis group exhibited a significantly lower Shannon diversity index than those from the non-fungal rhinosinusitis group (*t* test *p* = 0.029); (**b**) no significant difference in Shannon diversity was observed between fungal and non-fungal rhinosinusitis groups in terms of nasopharynx samples (*t* test *p* = 0.85); and (**c**) no significant difference in Shannon diversity was observed in samples collected from different anatomical sites (nasopharynx and middle meatus) between fungal rhinosinusitis (*p* = 0.11) and (**d**) nonfungal rhinosinusitis (*p* = 0.35) groups. FS: fungal rhinosinusitis; nFS: non-fungal rhinosinusitis; MM: middle meatus; NP: nasopharynx.

Figure 1 presents differences in Shannon diversity between the samples collected from different anatomical sites in fungal and non-fungal rhinosinusitis. As shown in Figure 1a, the Shannon diversity index observed for the fungal rhinosinusitis samples was significantly lower than that observed for the non-fungal rhinosinusitis samples collected from the middle meatus (lesion site). However, no significant difference was observed between nasopharynx samples collected from the fungal and nonfungal rhinosinusitis groups (Figure 1b). Additionally, Figure 1c,d illustrates no difference in Shannon diversity between middle meatus samples and nasopharynx samples in either the fungal or nonfungal rhinosinusitis group.

**Figure 2:**
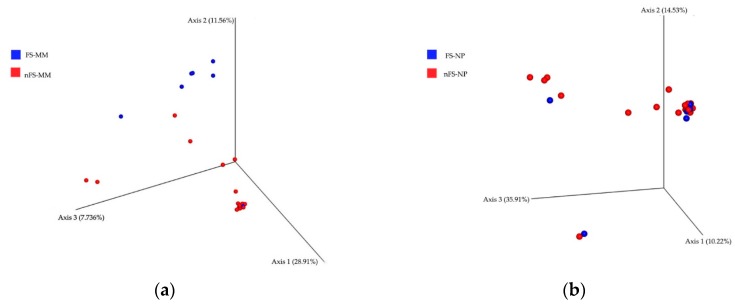
16S rRNA gene–based bacterial community compositions and intersubjective variability at the bacterial genus level were compared using the Bray–Curtis distance matrix. Points represent each individual and the relative similarity of members of a bacterial community—incorporating both presence/absence and relative abundance of bacterial community members—when compared with all other individuals (closer = more similar, farther apart = more dissimilar) [3]. (**a**) Middle meatus samples in the fungal rhinosinusitis group (blue symbols) were more closely clustered than those in the non-fungal group (red symbols). No other comparison showed similar clustering behavior as the nasopharyngeal samples (**b**) in the fungal rhinosinusitis group (blue symbols) and nonfungal rhinosinusitis group (red symbols).

As displayed in Figure 2, a principal component analysis was performed using the Bray–Curtis distance matrix to determine the relationships between various bacterial communities in the two groups. Fungal rhinosinusitis samples were more closely clustered than nonfungal samples (Figure 2a) and did not differ in other comparisons (Figure 2b).

**Figure 3:**
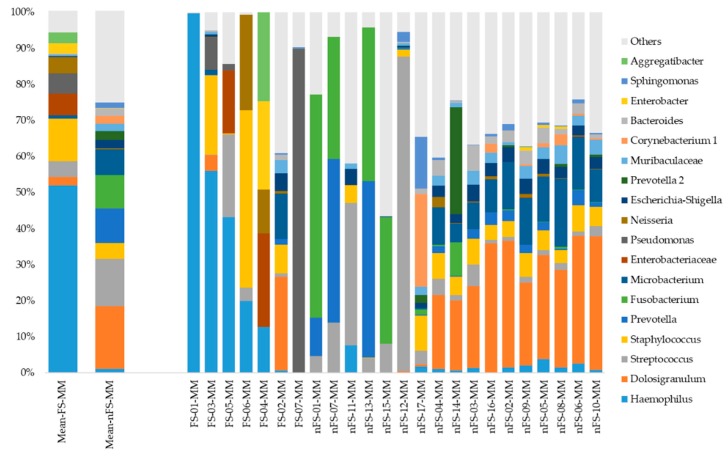
16S rRNA gene–based bacterial community composition and abundance data for middle meatus samples collected from patients with fungal rhinosinusitis (labeled with FS) and those with nonfungal rhinosinusitis (labeled with nFS).

Figure 3 illustrates the microbiota distribution (relative OTU composition) at the genus level in the middle meatus. Distribution of microbiota in samples collected from the nasopharyngeal or under different conditions are shown in Figure S1a,b,c. As shown in Figure 3, the *Haemophilus* genus was dominant in samples from the fungal rhinosinusitis group; by contrast, *Dolosigranulum* and *Streptococcus* were dominant in middle meatus samples from the non-fungal rhinosinusitis group.

Table 2 presents the dominant OTUs in middle meatus samples collected from fungal rhinosinusitis and nonfungal rhinosinusitis groups. *Pseudomonas* and *Haemophilus* were significantly dominant in the samples from the fungal rhinosinusitis group, and *Corynebacterium* and *Fusobacterium* were dominant in the samples from the nonfungal rhinosinusitis group.

**Figure 4:**
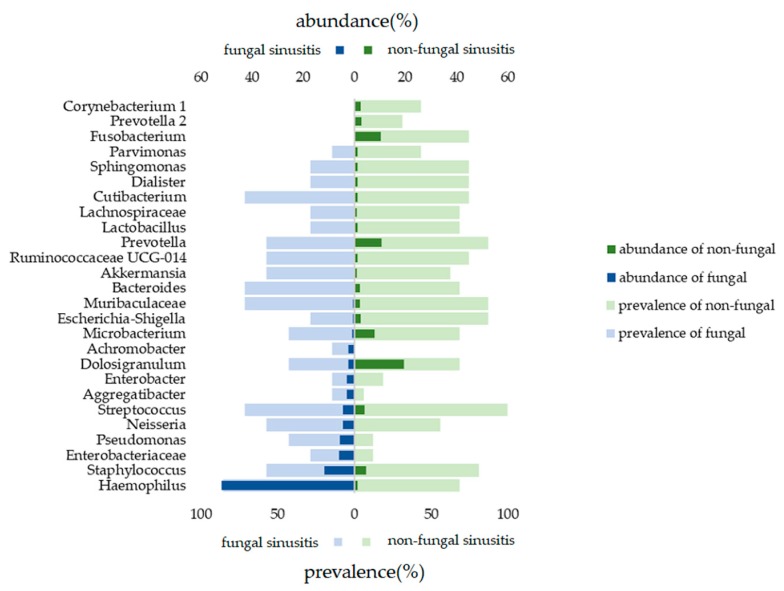
Abundance and prevalence of bacterial microbiota between middle meatus samples collected from fungal rhinosinusitis and nonfungal rhinosinusitis groups. Prevalence of a taxon was calculated by the number of samples containing the taxon divided by the total number of samples. The abundance of a taxon was calculated by the mean proportion of the taxon in samples. This figure includes only taxa with an average proportion of more than 1% in samples.

Figure 4 illustrates taxa with an average proportion of more than 1% in samples obtained from the middle meatus; the figure also presents the prevalence of such taxa. *Corynebacterium*, *Prevotella,* and *Fusobacterium* were detected only in nonfungal samples, and *Achromobacter* was detected only in fungal samples. *Haemophilus* was not only highly prevalent (85.7%) but also relatively abundant (51.8%) in fungal microbial communities. *Pseudomonas* was also highly prevalent and abundant in fungal microbial communities.

**Figure 5:**
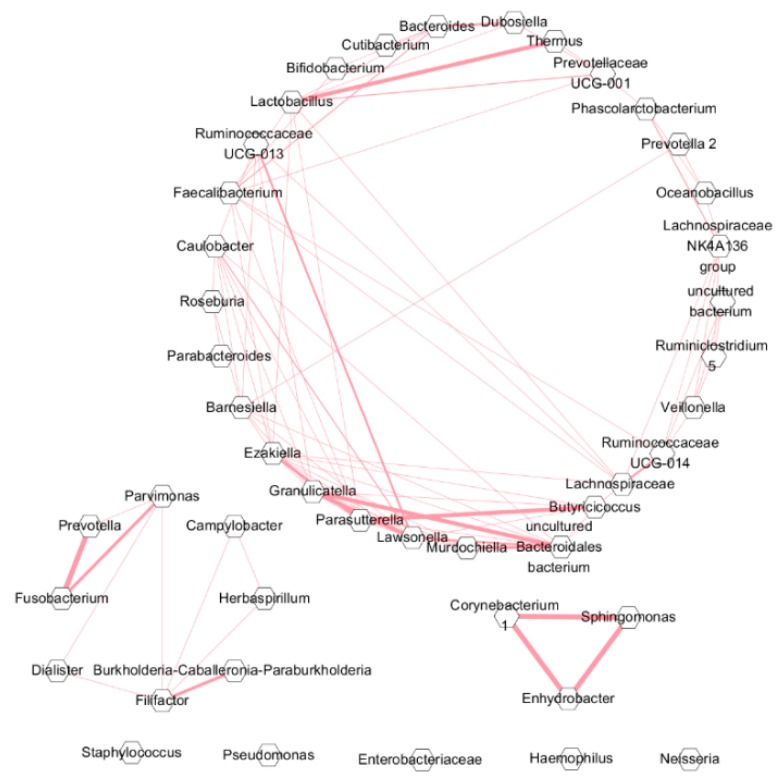
Correlation between microbiota in middle meatus samples from fungal and non-fungal rhinosinusitis groups. Correlations with a Pearson correlation coefficient greater than 0.6 were drawn with links using Cytoscape.

Figure 5 presents the correlation network. *Corynebacterium, Sphingomonas,* and *Enhydrobacter* were highly connected and positively correlated in fungal rhinosinusitis samples.

**Figure 6:**
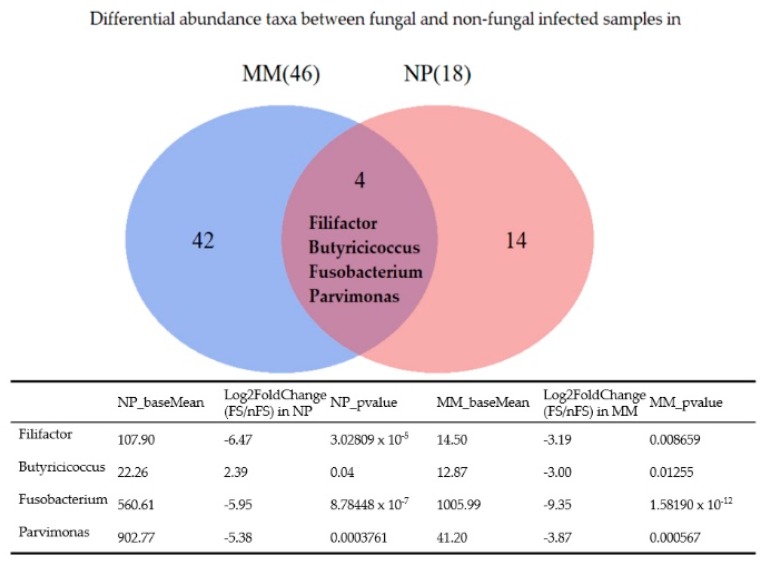
Venn diagram of differentially abundance taxa between fungal and non-fungal rhinosinusitis samples collected from the middle meatus and nasopharynx; four genera are shown at the intersection.

Figure 6 shows a Venn diagram of the differentially abundant taxa between fungal and nonfungal sinusitis samples obtained from the middle meatus and nasopharynx. *Fusobacterium*, *Filifactor,* and *Parvimonas* were less abundant in fungal sinusitis samples obtained from both regions. *Butyricicoccus* was more abundant in fungal rhinosinusitis samples obtained from the nasopharynx but less abundant in samples obtained from the middle meatus when compared with nonfungal rhinosinusitis samples.

## 4. Discussion

This study applied next generation sequencing to evaluate bacterial communities in different phenotypes of rhinosinusitis (fungal vs. nonfungal rhinosinusitis) and at different anatomical sites (the middle meatus vs. nasopharynx). The study included seven consecutive patients with fungal rhinosinusitis and 18 patients with nonfungal rhinosinusitis. *Aspergillus* was identified in all middle meatus samples from the fungal rhinosinusitis group but not in the non-fungal rhinosinusitis group. Compared with samples from the non-fungal rhinosinusitis group, dysbiosis was observed in middle meatus samples from the fungal rhinosinusitis group, as revealed by the Shannon test (*t* test *p* = 0.0029). However, no significant difference was noted between nasopharyngeal samples obtained from the fungal and non-fungal rhinosinusitis groups (*t* test *p* = 0.85). On the basis of these results, we can conclude that fungal sinusitis is a relatively localized disease because differences in bacterial microbiota were observed at only the lesion site (middle meatus) and not at the non-lesion site (nasopharynx), a finding that is compatible with the clinical features of fungal sinusitis. Furthermore, the prevalence and abundance of bacterial communities differed between fungal and non-fungal rhinosinusitis samples. *Corynebacterium* and *Fusobacterium* were detected in only nonfungal sinusitis samples. By contrast, *Haemophilus* and *Pseudomonas* were not only highly prevalent but also are abundant in fungal microbial communities. Surprisingly, *Haemophilus* even reaches nearly 99% of middle meatus microbiota in a fungal sinusitis sample (FS-01-MM), of which qualified DNA was extracted from a female patient with left fungal sinusitis. That patient had no previous endoscopic sinus surgery history or any other systemic disease.

Fungal rhinosinusitis, involving a spectrum of disease processes, varies in clinical presentation, histologic appearance, and biological significance [7]. The difference between fungal and non-fungal rhinosinusitis is based on the presence or absence of hyphae in histological findings. Additionally, according to pathological findings, fungal rhinosinusitis can be subdivided into invasive and noninvasive fungal sinusitis [7]. Notably, noninvasive fungal rhinosinusitis, particularly fungal balls, is commonly identified unilaterally in the maxillary sinus [7,8]; the reasons for this phenomenon are unknown. In our study, all seven cases of fungal rhinosinusitis were noninvasive (fungal balls). Furthermore, similar to the aforementioned findings of previous studies, all of them were unilateral and located in the maxillary sinus. Compared with fungal rhinosinusitis presentations, only 50% (nine of 18) of non-fungal rhinosinusitis presentations were unilateral; all of these non-fungal rhinosinusitis presentations were characterized by multi-sinus inflammation—extending to posterior sinus groups such as the sphenoid sinus and posterior ethmoid sinus—rather than single sinus inflammation. The varying range of sinus extension in fungal and non-fungal rhinosinusitis could lead to different results in the Shannon test. Although no significant difference was observed in the *t* test, the trend demonstrated the existence of bacterial microbiome diversity between fungal rhinosinusitis samples obtained from the nasopharynx and middle meatus (Figure 1c). By contrast, the bacterial microbiota observed in samples collected from the nasopharynx and middle meatus were similar in the nonfungal rhinosinusitis group (Figure 1d).

In fungal rhinosinusitis, the fungus colonizes the sinus, which could lead to fungal–bacterial interactions. Such fungal–bacterial interactions can be antagonistic, synergistic, commensal, or symbiotic and influence the physical and physiological characteristics such as the mutual morphology, behavior, and survival (including response to antimicrobial agents) characteristics [9,10]. Therefore, fungal–bacterial interactions could lead to bacterial microbiota dysbiosis in fungal rhinosinusitis, particularly at the lesion site (middle meatus; Figure 1a). However, nasopharyngeal samples showed no significant difference in bacterial microbiota diversity between the fungal and non-fungal rhinosinusitis groups (Figure 1b). The cause of the aforementioned results could be related to the site of fungal infection. In our study, fungal hyphae were found in only the middle meatus instead of the nasopharynx. Therefore, the fungal–bacterial interaction was relatively weak in the nasopharynx, even in the fungal rhinosinusitis groups.

In our study, *Aspergillus* was the predominant genus. *Aspergillus* hyphae were discovered in all middle meatus samples collected from the seven patients with fungal rhinosinusitis. By contrast, no fungal hyphae were discovered in the nasopharynx samples collected from the patients with fungal rhinosinusitis or nasopharynx/middle meatus samples collected from the patients with non-fungal rhinosinusitis. Similarly, Zhao et al. revealed that *Aspergillus* was the most common fungal microbiota in CRS and was not abundant in every CRS case [4]. They identified that only nine of 63 CRS cases were fungal groups with a relatively high ITS concentration [4], which is also similar to our results that fungal hyphae were not present in every rhinosinusitis sample. 

Previous studies have discovered inter-microbial interactions between *Aspergillus* and bacteria [11,12,13,14,15,16]. *Haemophilus influenzae* and *Haemophilus parainfluenzae* were reported in ethmoidal sinusitis samples that were analyzed through DNA sequencing using an Illumina/Solexa sequencing platform, and *Aspergillus oryzae* and *Aspergillus flavus* were the dominant fungal species [11]. *H. influenza* was also observed to be a possible coinfection with *A. flavus* in dual infectious brainstem encephalitis, which may be related to chronic fungal sinusitis [12]. In our study, *Haemophilus* was not only highly prevalent (85.7%) but also relatively abundant (51.8%) in fungal microbial communities. Yet, the microbiome results of sample FS-01-MM was unique. Notably, the OTUs of *Pseudomonas, Haemophilus, Enterobacteriaceae, Neisseria,* and *Staphylococcus* were found to be highly increased in the fungal sinusitis samples compared with the nonfungal sinusitis samples (Figure 4). The high residency and microaerobic tolerance of these nasal bacteria may contribute to that dominance. A culture-based study revealed that *Staphylococcus aureus* (31.9%), *Pseudomonas aeruginosa* (21.2%), and *Haemophilus* sp (8.5%) were common coinfection bacteria in patients with fungal rhinosinusitis [12]. Moreover, bacterial coinfection was observed to be abundant in the nasal swabs of patients with rhinosinusitis presented in the form of fungal balls (85.19%). *Staphylococcus sp.* and *Streptococcus* sp. were dominant in both fungal-culture-positive (18 and four cases, respectively) and fungal- culture- negative (23 and 10 cases, respectively) groups. However, notably, *Pseudomonas* sp. (10 cases) and *Klebsiella* sp. (seven cases) were also isolated in the fungal-culture-negative group [14]. Iron acquisition may play a major role in the antibiosis between nasal common resident bacteria and fungi. Previous research has reported that *P. aeruginosa* produces siderophores that inhibit *Aspergillus fumigatus*, and *A. fumigatus* siderophores protect against competition by *P. aeruginosa* [15,16]. Fungal infections do not seem to eradicate common bacterial rhinosinusitis pathogens. In this study, OTUs of *Corynebacterium* and *Fusobacterium* were significantly reduced (<2%) in the fungal group relative to the nonfungal group (Figure 4). *Corynebacterium* is a genus of aerobic bacteria whose environment would be affected by fungal growth. However, the reduction of the obligately anaerobic *Fusobacterium*, which is predominant in chronic rhinosinusitis [17], in the fungal group may be attributed to the interplay between bacteria and the fungi in a contact-dependent manner [18].

Many taxa were found to be differentially abundant between fungal and nonfungal rhinosinusitis samples collected from the middle meatus (Table 2). However, only *Fusobacterium*, *Filifactor,* and *Parvimonas* were more abundant in nonfungal sinusitis samples collected from both anatomical sites (middle meatus and nasopharynx), as illustrated by the Venn diagram (Figure 6). *Filifactor,* belonging to the phylum *Firmicutes,* is a gram-positive, slow-growing anaerobic bacterium [19]. Recent studies have demonstrated that *F. alocis* increases at the sites of periodontal disease and has synergistic interactions with other common periodontal bacteria, which leads to the colonization of pathogenic periodontal communities [20,21,22]. *Parvimonas*, a gram-positive anaerobic coccus, is another well-known oral pathogen and is associated with periodontitis in humans [19,23]. *Fusobacterium*, a gram-negative anaerobic bacterium, is numerically dominant in dental plaque biofilms and critical in the biofilm ecology and human infectious diseases [24]. *F. nucleatum* can also coaggregrate properties to transport periodontopathogenic bacteria [24]. Accordingly, *Filifactor*, *Parvimonas,* and *Fusobacterium* have all been found to be related to odontogenic infections. Therefore, we hypothesized that non-fungal rhinosinusitis could have a closer relationship with odontogenic infection or periodontitis compared with fungal rhinosinusitis.

This study has several limitations. First, compared with other phenotypes of sinusitis, fungal rhinosinusitis was relatively rare; therefore, we included seven consecutive patients with fungal sinusitis in this study. Second, the study did not evaluate fungal microbiota. Third, invasive fungal sinusitis was not included in this study. Forth, three patients (one fungal sinusitis and two non-fungal sinusitis) with revision endoscopic surgery history were included in this study, although there is no significant difference between the two groups (*p =* 0.865), but previous surgery could also be related to documented changes in bacterial composition and abundance in the middle meatus [25]. Accordingly, future research should include more patients and evaluate ITS fungal microbiota to comprehensively elucidate fungal sinusitis.

## 5. Conclusions

To the best of our knowledge, this is the first study to use NGS to investigate the difference in bacterial microbiota between patients with fungal rhinosinusitis and those with non-fungal rhinosinusitis. We revealed that bacterial microbiome dysbiosis was significantly apparent in patients with fungal rhinosinusitis compared with those with nonfungal rhinosinusitis. Furthermore, microbiome dysbiosis was discovered in only middle meatus samples and not in nasopharyngeal samples; this finding is compatible with the clinical characteristics of fungal sinusitis as limited and locally occupied sinus lesions. *Haemophilus* and *Pseudomonas* were more abundant in the fungal rhinosinusitis group, but *Fusobacterium* and *Corynebacterium* were more abundant in the nonfungal rhinosinusitis group; this could be associated with bacterial–fungal *(Aspergillus)* interactions. In the future, further research should be conducted to explore the characteristics of fungal rhinosinusitis to prevent sinusitis development, marking improved treatment.

## Figures and Tables

**Table 1:** Demographics of patients.

	Fungal Rhinosinusitis (*n* = 7)	Non-Fungal Rhinosinusitis (*n* = 18)	*p*-Value
Gender (M:F)	4:3	11:7	0.856
Asthma	0	0	NA
DM ^1^	0	2	1.00
Smoking	0	4	0.294
Previous FESS ^2^	1	2	0.826
Endoscope features			
Purulence	7	13	0.294
Polyp	2	11	0.202
Unilateral/Bilateral CT ^3^ features Discrete calcification Sample Origin: Nasopharynx Middle meatus	7/0 7 6 7	9/9 0 18 17	0.057 0.000

^1^ DM: Diabetes mellitus; ^2^ FESS: functional endoscopic sinus surgery; ^3^ CT: Computed tomography.

**Table 2:** Dominant operational taxonomic units (OTUs) in middle meatus samples.

Taxonomy	Mean Proportion-Fungal	Log2foldchange	*p*-Value
Pseudomonas	0.157621	7.041475	1.34 × 10^−16^
Fusobacterium	9.39 × 10^−5^	−10.4598	1.58 × 10^−12^
Enterobacteriaceae	0.112324	6.760324	2.39 × 10^−11^
Haemophilus	0.39881	3.39837	1.93 × 10^−9^
Corynebacterium 1	9.39 × 10^−5^	−8.62602	5.8 × 10^−7^
Faecalibacterium	0.001241	−4.01124	3.06 × 10^−6^
Lawsonella	9.39 × 10^−5^	−6.46435	1.04 × 10^−5^
Roseburia	9.39 × 10^−5^	−6.3614	1.09 × 10^−5^
Prevotella 2	9.39 × 10^−5^	−8.5811	1.85 × 10^−5^
Dialister	0.003497	−2.87925	2.44 × 10^−5^
Neisseria	0.077839	3.064623	2.71 × 10^−5^
Phascolarctobacterium	9.39 × 10^−5^	−5.8985	5.28 × 10^−5^
Bifidobacterium	9.39 × 10^−5^	−5.55629	8.48 × 10^−5^
Sphingomonas	0.002662	−3.92634	0.000283
Parasutterella	0.000142	-6.25574	0.000274
Parvimonas	0.000105	−7.56253	0.000576
Cutibacterium	0.004112	−2.95335	0.001021
Prevotellaceae UCG-001	9.39 × 10^−5^	−5.67206	0.000922
Barnesiella	9.39 × 10^−5^	−5.36435	0.001773
Prevotella	0.009641	−3.5138	0.002127
Campylobacter	0.002497	−2.78179	0.003202
Dubosiella	0.001719	−1.34162	0.003869
uncultured bacterium	9.39 × 10^−5^	−4.31437	0.004209
Herbaspirillum	0.001283	−0.91395	0.004913
Burkholderia-Caballeronia-Paraburkholderia	0.001427	−1.42577	0.005533
Murdochiella	0.000407	−3.49905	0.00606
Thermus	0.006022	0.12931	0.007197
Filifactor	9.39 × 10^−5^	−6.65602	0.008659
Caulobacter	9.39 × 10^−5^	−5.71	0.009229
Granulicatella	9.39 × 10^−5^	−5.01554	0.011858
Butyricicoccus	9.39 × 10^−5^	−5.86795	0.01255
Ruminococcaceae UCG-013	0.000228	−3.92258	0.012505
Parabacteroides	0.002514	−2.20143	0.01374
Oceanobacillus	0.000385	−3.95811	0.015384
Lactobacillus	0.008292	−1.74028	0.015927
Ezakiella	0.002612	−2.88786	0.017742
Lachnospiraceae	0.008553	−1.38552	0.02134
Bacteroides	0.009134	−2.6368	0.026386
uncultured Bacteroidales bacterium	0.000326	−5.18799	0.034265
Staphylococcus	0.151025	0.366581	0.036067
Lachnospiraceae NK4A136 group	0.004441	−1.25274	0.033673
Veillonella	0.006022	−2.41288	0.035876
Enhydrobacter	0.000119	−5.33978	0.035423
uncultured bacterium	0.010302	−1.14696	0.038908
Ruminiclostridium 5	0.00215	−1.34321	0.039208
Ruminococcaceae UCG-014	0.011234	−1.72203	0.04779

## Supplementary Materials

The following are available online at https://www.mdpi.com/2077-0383/8/11/1973/s1.
